# Rifaximin reduces the incidence of spontaneous bacterial peritonitis, variceal bleeding and all‐cause admissions in patients on the liver transplant waiting list

**DOI:** 10.1111/apt.15326

**Published:** 2019-06-06

**Authors:** Shayon Salehi, Thomas H. Tranah, Samuel Lim, Nigel Heaton, Michael Heneghan, Varuna Aluvihare, Vishal C. Patel, Debbie L. Shawcross

**Affiliations:** ^1^ Institute of Liver Studies and Transplantation King's College Hospital London UK; ^2^ Liver Sciences, Faculty of Life Sciences and Medicine, 1st Floor James Black Centre, School of Immunology and Microbial Sciences King's College London London UK

## Abstract

**Background:**

Rifaximin reduces the risk of overt hepatic encephalopathy (HE) and is associated with significant reductions in hospitalisations and 30‐day readmissions.

**Aim:**

To examine the outcomes of patients listed for liver transplantation with a diagnosis of HE on rifaximin compared to those naïve to the drug.

**Methods:**

Patient records of those listed for liver transplantation over a 2‐year period were retrospectively reviewed. Patients were included if they had at least two episodes of overt HE resulting in hospitalisation or were encephalopathic at the time of assessment.

**Results:**

Of the 622 patients listed for transplantation, 101 had HE. Sixty‐six patients were treated with rifaximin and 35 were naïve at listing. The use of concurrent lactulose was not significantly different between groups. Median MELD score was similar (15 [14‐16)] rifaximin‐treated and 16 [14‐18] rifaximin‐naïve). Patients on the waiting list treated with rifaximin had reduced all‐cause admissions, episodes of spontaneous bacterial peritonitis and variceal bleeding. Mean length of stay was 9 days (95% CI 6‐12) in the rifaximin‐treated group vs 14 (95% CI 7‐21) in the rifaximin‐naïve group. Multivariate regression analysis demonstrated that rifaximin was independently associated with an increase in average days to readmission (adjusted effect estimate 71, 95% CI 3‐140 days) and reduced likelihood of requirement for prioritisation on the waiting list (odds ratio 0.29; 95% CI 0.89‐0.93).

**Conclusion:**

Rifaximin prescribed for HE in patients listed for liver transplantation improved outcomes with significant reduction in admissions related to spontaneous bacterial peritonitis, ascites and variceal bleeding.

## 1 INTRODUCTION

The onset of advanced cirrhosis brings with it a catalogue of complications affecting multiple organ systems and includes hepatic encephalopathy (HE), variceal bleeding, ascites and a propensity to developing infections such as spontaneous bacterial peritonitis which can lead to the rapid onset of renal failure and metabolic disarray. Patients may progress to develop acute‐on‐chronic liver failure (ACLF) and the associated morbidity and mortality is high. These patients require frequent hospitalisation^1^ often necessitating high dependency or intensive care and present a significant healthcare and resource burden.^2^ Without access to liver transplantation, the outlook is bleak.^3^

The development of HE in both its covert^4^ and overt forms^3^, ^5^ confers a poor prognosis. The 1‐year mortality following a diagnosis of cirrhosis in the absence of any evidence of decompensation is approximately 17% but approaches 64% following the development of overt HE.^6^ The non‐absorbable antibiotic rifaximin reduces the risk of recurrence of overt HE and the need for hospitalisation.^7^ Whilst the specific mechanism of action of rifaximin remains to be elucidated, it has been postulated to reduce circulating levels of gut‐derived endotoxins^8^ resulting from bacterial translocation.^9^ In clinical practice, treatment with rifaximin has been associated with significant reductions in hospitalisation, bed days (including critical care), emergency department attendances and 30‐day readmissions.^10^, ^11^ However, there is a paucity of data on the impact of rifaximin on outcomes in patients with end‐stage liver disease.

The aim of this study was to examine the outcomes of patients with advanced cirrhosis listed for liver transplantation at a large tertiary referral centre with a diagnosis of HE at listing treated with rifaximin compared to those naïve to the drug. The primary objective was to compare the frequency and duration of all‐cause emergency hospital admissions. Secondary objectives included incidence of infection and admissions related to complications of cirrhosis, admissions to critical care, requirement for prioritisation on the waiting list and mortality.

## 2 MATERIALS AND METHODS

### 2.1 Study design and setting

The patient records of 622 patients with confirmed cirrhosis (the diagnosis of cirrhosis was confirmed by a combination of a least 2 modalities: clinical, biochemical, radiological and histopathological) who were listed for liver transplantation at King's College Hospital NHS Foundation Trust over a 2‐year period [1st January 2014 – 31st January 2016] were retrospectively reviewed.

### 2.2 Participants

Patients were included if they had at least two historic episodes of overt HE resulting in hospitalisation or were overtly encephalopathic at the time of assessment. Patients under the age of 18 were excluded from the study.

### 2.3 Data collection

Information collected included patient demographics, aetiology of liver disease, Child Pugh Turcotte score,^12^ Model for End‐stage Liver Disease (MELD) score^13^ at the time of assessments, United Kingdom End‐stage Liver Disease (UKELD) score, maximum grade of HE (defined using the West Haven criteria),^14^ blood ammonia concentration (venous), concurrent lactulose therapy, medical co‐morbidities, emergency admission whilst on the waiting list including admissions to high dependency and intensive care beds, requirement for prioritisation (UKELD score ≥63), duration on the waiting list (days) and mortality on the waiting list. Elective admissions, such as for large volume paracentesis were excluded unless the paracentesis was complicated (defined as necessitating a hospital admission for greater than 24 hours) which may occur for example due to spontaneous bacterial peritonitis, acute kidney injury or electrolyte disturbance.

### 2.4 Statistical methods

The primary outcome was defined as the number of days to all cause readmission on the transplant waiting list. Secondary outcomes evaluated included requirement for prioritisation, hospital admissions with sepsis, variceal bleeding, ascites and hepatic encephalopathy, length of hospital stay, intensive care admissions, length of intensive care stay and mortality on the waiting list. Normality testing was undertaken on all continuous clinical and biochemical data using the D'Agnostino‐Pearson omnibus normality test. Normally distributed data are presented as mean with 95% CIs and non‐normally distributed data as median and interquartile range. Univariate statistical analysis of non‐normally distributed unpaired data was completed using the Mann–Whitney test and analysis of normally distributed data using unpaired *t* tests with Welch's correction assuming unequal standard deviations. Categorical variables are expressed as number and proportion and compared using the *X* ^2^ test or Fisher's exact test, as appropriate. Hospital admission data were presented as annualised rates. Multivariate linear regression analysis was performed on admission and complication data (related to sepsis, acute variceal hemorrhage, encephalopathy and complications of ascites) identified as the dependent variable. Binary logistic regression analysis was subsequently performed with requirement for prioritisation on the transplant waiting list (yes/no) as the dependent variable. Forward selection was used to select independent variables within the regression models with *P* < 0.1; we also included variables considered by subject knowledge or literature (ie lactulose use and MELD score) to be associated with hospital readmission in end‐stage chronic liver disease. Univariate and regression analyses were performed using IBM spss Statistics 24 for Mac. A *P* < 0.05 was considered to be statistically significant.

## 3 RESULTS

### 3.1 Patient demographics

Of the 622 adult patients listed for transplantation, 101 were listed with HE. Sixty‐six patients were treated with rifaximin and 35 were naïve. There was a male preponderance in both groups and alcohol‐related liver disease was the most common aetiology. The rifaximin‐treated group was marginally older mean age 55 vs 49 (mean difference 5.85 years [95% CI 0.72‐10.98]). Organ severity scores were similar; mean MELD score was 15 (95% CI 14‐16) in the rifaximin cohort and 16 (95% CI 14‐18) in the naïve [mean difference −1.1 (95% CI −3.49‐1.38)]. Mean Child‐Pugh Turcotte score was 10 (95% CI 9.4‐10.2) in the rifaximin cohort and 10 (95% CI 9.7‐10.9) in the naïve (mean difference −0.52 [95% CI −1.24‐0.21]). 82% of those on rifaximin and 71% of those on placebo were on concurrent lactulose therapy (*P* = 0.26). Fifteen percent of the rifaximin cohort (10/66) were on ciprofloxacin prophylaxis (500 mg once daily) and 20% of the rifaximin‐naive cohort (7/35) were on ciprofloxacin prophylaxis (500 mg once daily) for primary or secondary prophylaxis of spontaneous bacterial peritonitis; this was not statistically significantly different between the groups (*P* = 0.58). The mean duration on the waiting list, the maximum grade of encephalopathy at listing and the baseline venous ammonia levels did not differ between those on rifaximin and those naïve to the drug. Body Mass Index (BMI) and the number of medical co‐morbidities at the time of transplant assessment was similar across the treatment groups. The patient demographics are summarised in Table 1.

**Table 1:** Baseline patient demographics at transplant listing

Variable	Rifaximin treated n = 66	Rifaximin naïve n = 35	*P* value
Age [mean (95% CI)]	55 (52‐58)	49 (45‐53)	0.007
Male gender (%)	48 (73)	22 (63)	ns
BMI [mean (95% CI) kg/m^2^]	27.7 (26.3‐29.1)	26.0 (24.6‐27.4)	ns
Concurrent lactulose n (%)	54 (82)	25 (71)	ns
Duration on waiting list [mean (95% CI)]	185 (149‐221)	166 (109‐223)	ns
Maximum grade of HE [mean (95% CI)]	2 (1.7‐2.3)	2 (1.8‐2.2)	ns
Organ Severity Score			
MELD score [mean (95% CI)]	15 (14‐16)	16 (14‐18)	ns
UKELD score [mean (95% CI)]	56 (55‐57)	56 (55‐58)	ns
Child‐Pugh‐Turcotte Score [mean (95% CI)]	10 (9.6‐10.4)	10 (9.4‐10.6)	ns
Aetiology of Liver Disease			
Alcohol‐related cirrhosis n (%)	30 (46)	11 (31)	ns
NASH cirrhosis n (%)	2 (3)	2 (5)	ns
HCV cirrhosis n (%)	11 (17)	7 (19)	ns
HBV cirrhosis n (%)	0 (0)	1 (3)	ns
Autoimmune cirrhosis n (%)	12 (19)	9 (24)	ns
HCC n (%)	7 (11)	1 (3)	ns
Cryptogenic cirrhosis n (%)	6 (9)	4 (11)	ns

Abbreviations: CI: confidence interval; HE: hepatic encephalopathy; NASH: non‐alcoholic steatohepatitis; HBV: hepatitis B; HCV: hepatitis C; HCC: hepatocellular carcinoma; MELD: model for end‐stage liver disease; UKELD: UK end‐stage liver disease.

Spontaneous bacterial peritonitis and urinary tract infections are the commonest bacterial infections complicating cirrhosis‐associated immune dysfunction.

### 3.2 Clinical outcomes

On univariate analysis, rifaximin‐treated patients had reduced all‐cause admissions (elective admissions were excluded) on the waiting list 2.75 vs 6.30 (mean difference −3.55 [95% CI −6.545 to −0.56] admissions per year). Admissions with episodes of sepsis per year were similar between groups 0.84 vs 1.81 (mean difference −0.97 [95% CI −2.27‐0.33] admissions per year). Admissions related to complications of large volume ascites including spontaneous bacterial peritonitis, hepatorenal syndrome and metabolic disarray were reduced in the rifaximin‐treated patients mean 0.77 vs 2.47 (mean difference −1.70 [95% CI −2.99 to −0.41] admissions per year). Rifaximin‐treated patients had a reduced requirement for admission with acute variceal bleeding per year mean 0.14 vs 1.03 [mean difference −0.89 (95% CI −1.60 to −0.18) admissions per year]. Patients were less likely to warrant prioritisation on the transplant waiting list (defined during this study as having a UKELD score ≥63); odds ratio 0.34 (95% CI 013‐0.90).

**Figure 1:**
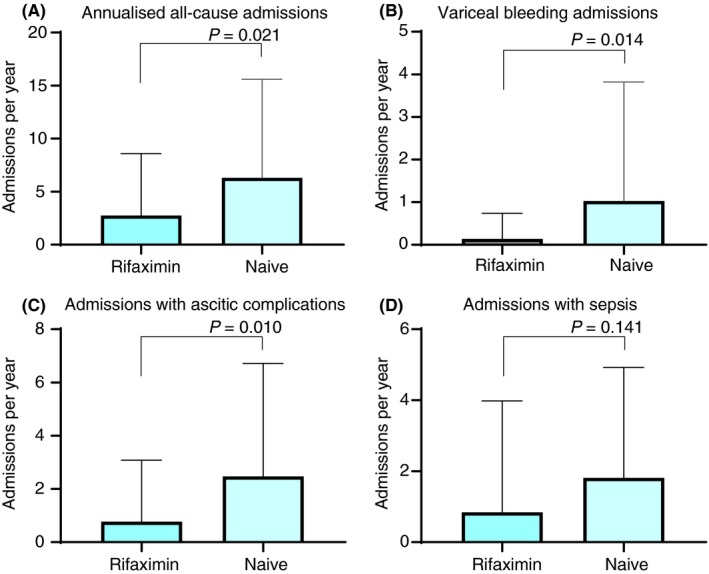
A, All‐cause admissions per year on the liver transplant waiting list (mean and standard deviation). B, Admissions with variceal bleeding per year on the liver transplant waiting list (mean and standard deviation). C, Admissions with complications of ascites (elective admissions for large volume paracentesis excluded) including spontaneous bacterial peritonitis, hepatorenal syndrome and metabolic disarray (mean and standard deviation). D, Admissions with sepsis per year on the liver transplant waiting list (mean and standard deviation)

Mean length of hospital stay (8.69 vs 14.43 days, mean difference −5.74 [95% CI −12.23 to 0.76] days) and mean length of intensive care unit stay (1.09 vs 2.49 days, mean difference −1.40 [95% CI −3.76 to 0.97] days) were not significantly different in rifaximin‐treated and rifaximin‐naïve groups. Interestingly, admissions with acute overt HE did not significantly differ and neither was mortality on the waiting list impacted upon despite the reduced rate of variceal bleeding, complications of ascites and all‐cause hospitalisation in the rifaximin‐treated cohort. The overall waiting list mortality in this study was 13.86%. Clinical outcomes are summarised in Table 2 and Figure 1.

**Table 2:** Clinical outcomes from univariate and multivariate analyses comparing rifaximin‐treated and rifaximin‐naïve patients on the liver transplant waiting list.

Outcome	Univariate analysis[unadjusted effect estimate (95% CI), *P* value]	Multivariate analysis [confounder‐adjusted effect estimate (95% CI), *P* value]
All‐cause admissions/year	−**3.55 (‐6.55 – ‐0.55), *P*=0.021**	−**3.10 (‐6.00 – ‐0.20), *P*=0.037**
Days to readmission	**+82 (48 – 117), *P*=0.025**	**+71 (3 – 139), *P*=0.040**
Admissions with sepsis/year	−0.97 (‐2.27 – 0.33), *P*=ns	−0.49 (‐1.75 – 0.98), *P*=ns
Admissions with complications of ascites including SBP/year	−**1.70 (‐3.00 – ‐0.4), *P*=0.010**	−**1.77 (‐3.07 – ‐0.47), *P*=0.008**
Admissions with acute variceal bleeding/year	−**0.89 (‐1.59 – ‐0.19), *P*=0.014**	−**0.81 (‐1.52 – ‐0.10), *P*=0.026**
Admissions with overt hepatic encephalopathy/year	−0.01 (‐0.81 – 0.79), *P*=ns	−0.07 (‐0.95 – 0.81), *P*=ns
Length of hospital stay (days)	−5.74 (‐12.5 – 1.06), *P*=ns	−6.35 (‐12.85 – 0.15), *P*=ns
Intensive care admissions/year	−0.46 (‐1.66 – 0.74), *P*=ns	−0.04 (‐1.18 – 1.10), *P*=ns
Length of intensive care stay (days)	−1.40 (‐3.80 – 1.20), *P*=ns	−1.15 (‐3.48 – 1.18), *P*=ns
Requirement for prioritisation on the waiting list (odds ratio)	**0.34 (0.13 – 0.90), *P*=0.03**	**0.29 (0.09 – 0.93), *P*=0.037**
Mortality on the waiting list (odds ratio)	0.66 (0.21 – 2.10), *P*=ns	0.40 (0.11 – 1.48), *P*=ns

Multivariate linear regression analysis with days to readmission (related to complications of ascites, variceal bleeding, hepatic encephalopathy or sepsis) defined as the dependent variable demonstrated that rifaximin treatment was independently associated with increased length to all‐cause readmission whilst on the liver transplant waiting list; adjusted effect estimate 71 (95% CI 3‐140 days) when adjusting for age, sex, BMI, disease severity score and concomitant lactulose use. Similarly, rifaximin use was independently associated with a significant reduction in the number of hospital admissions relating to variceal bleeding, complications of ascites and all‐cause hospital readmission (Table 2). Binary logistic regression was performed to assess requirement for prioritisation (UKELD ≥63) on the liver transplant waiting list and demonstrated that rifaximin treatment was independently associated with a lower likelihood of requirement for prioritisation (odds ratio 0.29 [95% CI 0.09‐0.93]) when adjusting for age, sex, BMI, disease severity score and concomitant lactulose use.

Of the 31 patients who were admitted due to suspected sepsis, a source of infection was only confirmed in cultures in 12 (38.7%) patients (13/66 [19.7%] on rifaximin vs 18/35 [51.4%] in those who were not treated with rifaximin; *P* = 0.016). This included three episodes of pneumonia, three episodes of spontaneous bacterial peritonitis, three episodes of bacteremia, two cases of cellulitis, one case of infective endocarditis and one case with a breast abscess. Organisms cultured included vancomycin‐resistant enterococcus, escheria coli, klebsiella pneumoniae, streptococcus viridans, coagulase negative staphylococcus aureus and *Clostridium difficile* (rifaximin‐naïve patient). Within our cohort, there were no documented cases of *C difficile* infection in patients taking rifaximin.

## 4 DISCUSSION

This retrospective ‘real‐world' cohort study examining the liver transplant waiting list outcomes of 101 patients listed with overt HE shows that those who were prescribed rifaximin for the recurrence of overt HE had improved outcomes on the waiting list with a significant reduction in all‐cause hospital admissions, an increased time to hospital readmission and reduced requirement for listing prioritisation compared to those naïve to rifaximin.

There is now a robust evidence base to support rifaximin as a beneficial adjunctive therapy to lactulose in the prevention of recurrent episodes of overt HE,^7^ reducing the risk of hospitalisation and lowering inpatient length of stay and healthcare resource utilisation.^10^, ^11^ Furthermore, it has been shown to improve health‐related quality of life in patients with cirrhosis and overt HE in remission^15^ and has been demonstrated to be safe and well tolerated in patients for long‐term maintenance of remission from overt HE.^16^ However, ‘real world' data on the impact of rifaximin on the outcomes of patients with advanced cirrhosis remains sparse and there are no data on its impact on the outcomes of patients listed for liver transplantation. Indeed, the pivotal Bass trial^7^ excluded patients with a MELD score of >25 with one‐fifth of the included patients having a MELD score of ≤10, two‐thirds with a MELD score between 11 and 18 and <10% with a MELD between 19 and 24. We therefore set out to determine the impact of treatment with rifaximin in patients listed with overt HE whilst they were on the liver transplant waiting list at a large UK transplant centre.

One of the strengths of this study was that whilst a large proportion of the patients included in this study had alcohol‐related cirrhosis because they had been abstinent for at least 6 months prior to listing, active alcohol intake was not a confounder in this study as it has been in other ‘real‐world' studies^10^, ^11^ where it was difficult to determine if abstinence from alcohol or rifaximin was the major driver in the improved outcomes in this cohort of patients.

Patients with advanced cirrhosis are susceptible to unplanned emergency hospitalisations for a variety of reasons, including susceptibility to infection,^17^ overt HE,^5^ acute kidney injury, acute variceal bleeding, electrolyte disturbance, large volume ascites, spontaneous bacterial peritonitis, falls, malnutrition and sarcopenia. These patients frequently progress to requiring high dependency or intensive care support^2^, ^18^ and may at any time progress to developing ACLF.^19^, ^20^

Infection is the leading cause of death in patients with end‐stage liver disease and confers a fourfold increased mortality compared to non‐infected patients; 30% of patients die within 1 month after infection and another 30% die by 1 year with spontaneous bacterial peritonitis and urinary tract infections representing the commonest bacterial infections complicating cirrhosis.^21^ Systemic inflammation and infection are also major drivers of episodes of HE^22^, ^23^ and have been implicated in patients admitted with advanced HE (grades 3 and 4) regardless of blood ammonia levels and MELD score.^24^ Systemic inflammation is also likely to be the single biggest driver for the development of ACLF. In the CANONIC study patients with acute decompensation of cirrhosis without ACLF showed very high baseline levels of inflammatory cytokines, markers of systemic oxidative stress and circulatory dysfunction. Moreover, patients with ACLF showed significantly higher levels of these markers than those without ACLF.^25^ It therefore follows that if patients in this study treated with rifaximin have reduced all‐cause unplanned hospitalisations and episodes of spontaneous bacterial peritonitis that rifaximin is reducing systemic inflammation and/or the susceptibility to developing infection.^26^ Indeed, it has been shown that rifaximin does reduce systemic endotoxin levels and this is likely to be linked to a change in the function rather than composition of the gut microbiome which we know exhibits dysbiosis in patients with advanced cirrhosis.^8^, ^27^ Indeed, a recent systematic review and meta‐analysis examining the impact of rifaximin on the development of spontaneous bacterial peritonitis showed that rifaximin may be effective in preventing spontaneous bacterial peritonitis in patients with cirrhosis and ascites compared to systemically absorbed antibiotics and compared to placebo.^9^ Another retrospective study examining 145 patients with cirrhosis showed rifaximin treatment was significantly associated with prolonged overall survival and reduced risks of spontaneous bacterial peritonitis, variceal bleeding and recurrent HE.^28^ A further randomised study of rifaximin vs placebo also showed that rifaximin prevented the development of hepatorenal syndrome which in many cases develops in association with spontaneous bacterial peritonitis.^29^ Our cohort of transplant‐listed patients with decompensated cirrhosis corroborates these findings with an associated reduction in hospital readmission with complications of ascites, including spontaneous bacterial peritonitis and hepatorenal syndrome independently associated with rifaximin use.

Our data also show that rifaximin independently led to a significant reduction in episodes of acute variceal bleeding on the transplant waiting list. Patients with cirrhosis and portal hypertension listed for transplantation invariably have severe portosystemic shunting with reverse flow in the portal vein and recanalisation of the umbilical vein. Furthermore, they have small bowel bacterial overgrowth, gut dysbiosis and increased gut permeability all contributing to bacterial translocation, endotoxemia and systemic inflammation.^30^, ^31^ This pathological process has been demonstrated to alter the hemodynamic circulation and could increase portal pressure. Rifaximin may therefore provide a therapeutic option to prevent portal hypertension‐related bleeding by reducing bacterial translocation and endotoxin levels. However, a recent small randomised controlled trial reported that 4 weeks of treatment with rifaximin did not reduce the hepatic venous pressure gradient or improve systemic hemodynamics in patients with cirrhosis and ascites. Furthermore, rifaximin did not affect glomerular filtration rate or levels of vasoactive hormones.^32^

Surprisingly, patients treated with rifaximin did not have a reduction in emergency encephalopathy‐related admissions per se. However, overall the absolute mean number of admissions attributed specifically to HE per year was low (1.00 [95% CI 0.46‐1.54] admissions per year in the rifaximin group vs 0.98 [95% CI 0.25‐1.73] in the naïve group) and patients listed for liver transplantation for HE invariably had severe and treatment‐refractory encephalopathy. It is also important to say that patients with admissions for falls, sepsis and bleeding often developed HE during that admission so a reduction in all‐cause admissions on rifaximin indirectly reduced the likelihood of developing HE. There was also no difference in mortality although overall the transplant waiting list mortality in this cohort was low at 13.89% so more patients would need to have been included to detect a mortality difference. Nevertheless, patients who deteriorate whilst on the waiting list may require prioritisation (in this study this was when their UKELD score reached 63 or above). Patients treated with rifaximin were less likely to require the need for prioritisation in this study and therefore this may have also had a bearing on any perceivable mortality difference. Where this study falls short, however, is that we were unable to determine in many cases the precise duration of rifaximin therapy prior to being referred and listed for transplantation as many patients were tertiary referrals from other centres where we could not access this information. There is no doubt that some smaller centres at that time did not have the resources or access to prescribe the drug which was licensed in January 2013 in the UK and approved by NICE in March 2014.

This study revealed an independent association of rifaximin use with a mean increase of 71 days to hospital readmission with 3.1 admissions per year avoided with a significant impact on healthcare resource utilisation. We did not observe a significant difference in hospital bed days, 8 in the rifaximin group vs 14 in the naïve group (mean effect estimate −6 [95% CI −12.23 to 0.76] days) and intensive care bed days, 1 in the rifaximin group vs 2 (mean effect estimate −1.40 [95% CI −3.76 to 0.97] days) in the naïve group. Moreover, recently published ‘real‐world' studies have shown that treatment with rifaximin was associated with a reduction in length of hospital stay and provides good value for money in terms of health economics and resource utilisation.^10^, ^11^

In summary, rifaximin prescribed for the recurrence of overt HE in patients listed for liver transplantation improved outcomes on the waiting list with a significant reduction in hospital admissions related to decompensation, variceal bleeding and complications of ascites. There was a reduced requirement for prioritisation of patients on the waiting list in patients treated with rifaximin and an increased time to hospital readmission. This study provides ‘real‐world' data that demonstrates the potential value of rifaximin in reducing hospital admissions and length of stay within the advanced cirrhotic population awaiting liver transplantation. At this point, however, the data should not be interpreted as a reason to change clinical practice but should act as a catalyst for further prospective studies in this patient group.

## AUTHORSHIP

*Guarantor of the article*: Debbie L. Shawcross.

*Author contributions*: DLS designed the study. SS and SL undertook data collection and performed the initial statistical analyses which were reviewed by THT who led on the final manuscript revision and statistical review with DLS. The manuscript was drafted by SS and extensively revised by THT and DLS. MH, VA and NH critically revised the manuscript and all authors approved the final submitted manuscript.
